# Supplementary material for: Identification of a Virulent Newcastle Disease Virus Strain Isolated from Pigeons (Columbia livia) in Northeastern Brazil Using Next-Generation Genome Sequencing
Source: Viruses. 2022 Jul 21;14(7):1579. doi: 10.3390/v14071579 (PMC9319777; doi:10.3390/v14071579)
Supplement: Supplementary file 1 [file viruses-14-01579-s001.zip › Supplementary Results_Proof.pdf]

Supplementary Results

Sick animals were collected and transported to the Laboratory of Animal Diagnosis, Federal Rural University of Pernambuco, Recife/PE, symptoms were registered and the animals were photographed. As illustrated in supplementary figure 1 the animals presented with neurological symptoms.

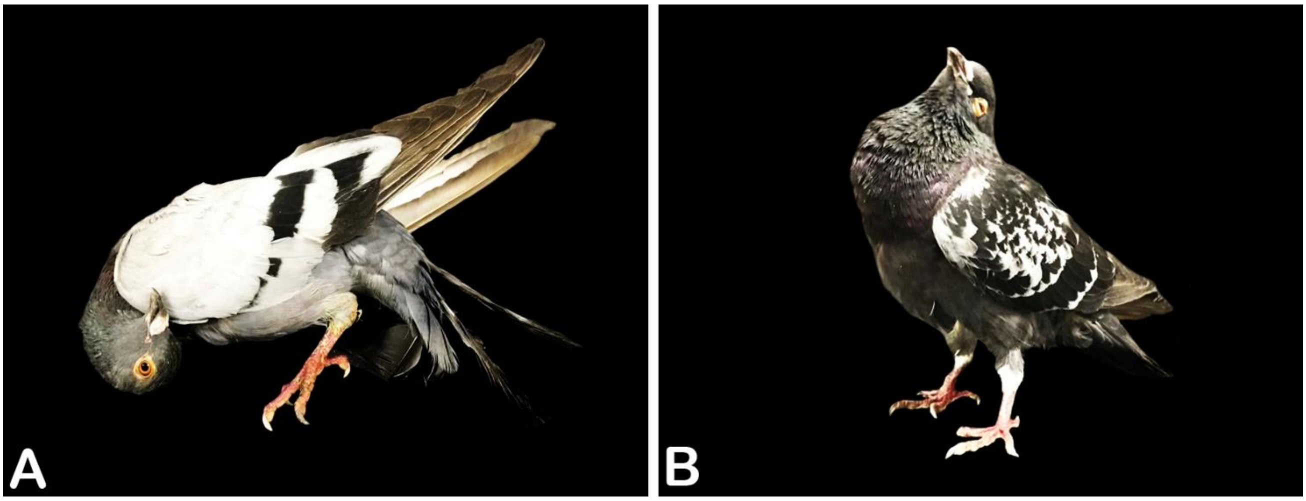

Figure S1. Free-living pigeons (*Columbia livia*) symptoms observed. **(A)** a bird presenting motor incoordination and torticollis **(B)** A bird with lethargy and opisthotonus.

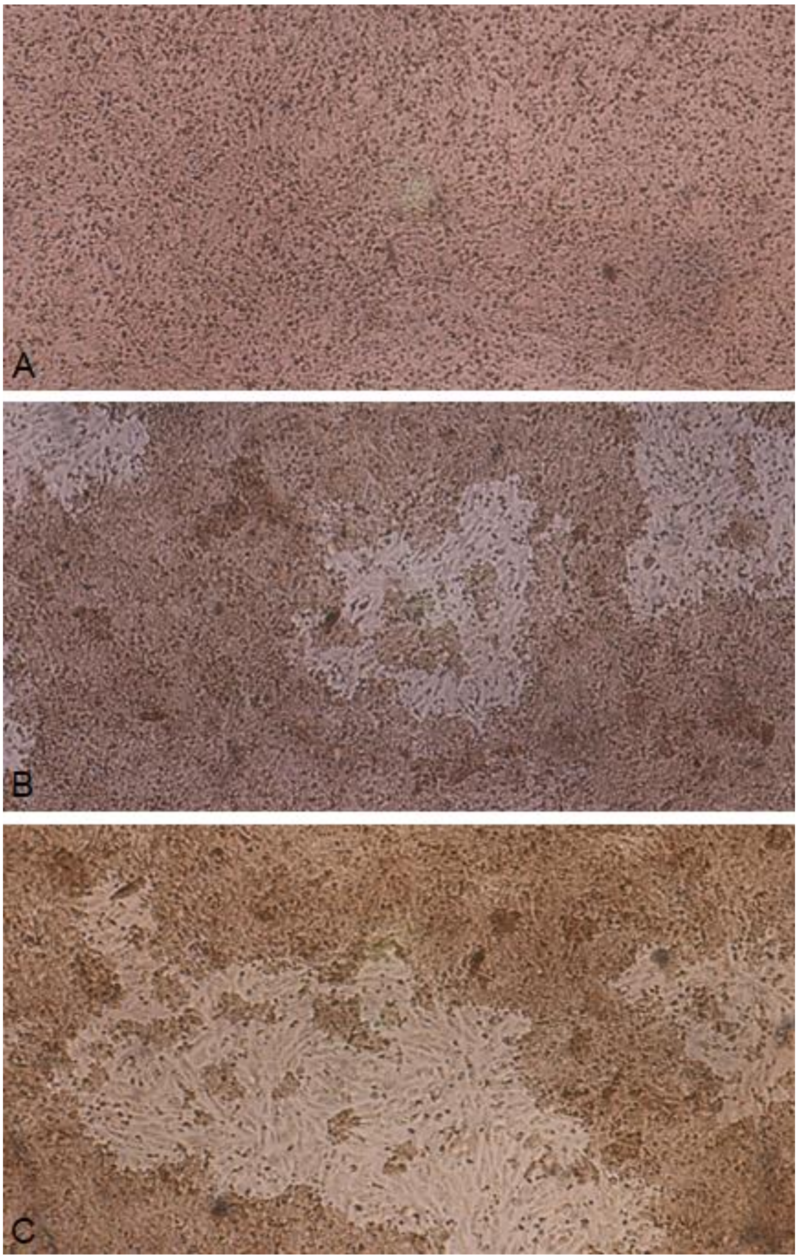

Figure S2. Cytopathic effect of NDV virus isolation on Vero E6 cell monolayers. **A** – Vero E6 cells not infected (control cells) presenting a regular morphology and absence of cell detachment; **B** – Vero E6 cells inoculated with homogenized brain samples from animal MP003 showing extensive cell detachment (suggestive of cell death) at 5 days after inoculation; **C** – Vero E6 cells inoculated with kidney samples from animal MP003 at 6 days after inoculation.
